# Standardizing the haemoglobin glycation index

**DOI:** 10.1002/edm2.299

**Published:** 2021-09-23

**Authors:** James M. Hempe, Shengping Yang, Shuqian Liu, Daniel S. Hsia

**Affiliations:** ^1^ Department of Pediatrics Louisiana State University Health Sciences Center New Orleans Louisiana USA; ^2^ Pennington Biomedical Research Center Baton Rouge Louisiana USA; ^3^ Tulane University School of Public Health and Tropical Medicine New Orleans Louisiana USA

**Keywords:** diabetes, haemoglobin glycation index, HbA1c, NHANES

## Abstract

**Aims:**

A high haemoglobin glycation index (HGI) is associated with greater risk for hypoglycaemia and chronic vascular disease. Standardizing how the HGI is calculated would normalize results between research studies and hospital laboratories and facilitate the clinical use of HGI for assessing risk.

**Methods:**

The HGI is the difference between an observed HbA1c and a predicted HbA1c obtained by inserting fasting plasma glucose (FPG) into a regression equation describing the linear relationship between FPG and HbA1c in a reference population. We used data from the 2005–2016 U.S. National Health and Nutrition Examination Survey (NHANES) to identify a reference population of 18,675 diabetes treatment–naïve adults without self‐reported diabetes. The reference population regression equation (predicted HbA1c = 0.024 FPG + 3.1) was then used to calculate the HGI and divide participants into low (<−0.150), moderate (−0.150 to <0.150) and high (≥0.150) HGI subgroups. Diabetes status was classified by OGTTs.

**Results:**

As previously reported in multiple studies, a high HGI was associated with black race independent of diabetes status, and with older age, higher BMI and higher CRP in normal and prediabetic but not diabetic participants. The mean HGI was 0.6% higher in self‐reported diabetic adults. The HGI was not associated with plasma insulin, HOMA‐IR or 2 h OGTT in participants classified as normal, prediabetic or diabetic.

**Conclusions:**

The regression equation derived from this demographically diverse diabetes treatment–naïve adult NHANES reference population is suitable for standardizing how the HGI is calculated for both clinical use and in research to mechanistically explain population variation in the HGI and why a high HGI is associated with greater risk for chronic vascular disease.

## Graphical abstract

The lack of standardization in calculating hemoglobin glycation index (HGI) in diverse populations is a significant barrier to HGI research and its possible clinical use. A simple linear regression equation using fasting glucose and HbA1c derived from the demographically diverse treatment naïve adult NHANES reference population is proposed to standardize the calculation of HGI. Standardizing the calculation of HGI will facilitate research into understanding why some people have discrepant HbA1c levels despite similar blood glucose concentrations and why a high HGI phenotype is associated with greater risk for chronic vascular disease.

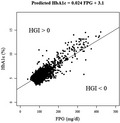

**What's New?**

- The lack of standardization in calculating the haemoglobin glycation index (HGI) in demographically diverse populations is a significant barrier to its clinical use as a measure of bias in the quantitative relationship between HbA1c and blood glucose concentration.
- A simple linear regression equation using fasting plasma glucose (FPG) and HbA1c derived from a demographically diverse diabetes treatment–naïve adult NHANES reference population is proposed to standardize HGI calculation.
- Standardizing how the HGI is calculated will facilitate research into understanding why some people have consistently higher or lower than average HbA1c levels than other people with similar blood glucose concentrations, and why a high HGI phenotype (i.e., higher HbA1c than predicted by FPG) is associated with greater risk for chronic vascular disease.

## 1 INTRODUCTION

The haemoglobin glycation index (HGI) is the difference between an individual's observed haemoglobin A1c (HbA1c) and a predicted HbA1c obtained by inserting a date‐matched blood glucose measurement into a linear regression equation describing the quantitative relationship between HbA1c and blood glucose in a reference population.^1^ As such, the HGI measures bias in the quantitative relationship between HbA1c and blood glucose concentration. Individuals with a low or high HGI have HbA1c levels that tend to be consistently lower or higher than average, respectively, than other people with similar blood glucose concentrations. Multiple clinical studies confirm that a high HGI (i.e., higher HbA1c than predicted by blood glucose) is associated with greater risk for chronic vascular disease in normal,^2^, ^3^, ^4^, ^5^ prediabetic,^6^ type 1 diabetic^7^ and type 2 diabetic^8^, ^9^, ^10^, ^11^ study populations. A high HGI has also been repeatedly associated with greater risk for iatrogenic (caused by medical intervention) hypoglycaemia in people with diabetes.^8^, ^9^, ^12^, ^13^, ^14^ Comparing HGI results between studies is confounded, however, by large interstudy variation in the slopes and intercepts of the linear regression equations used to calculate HGI; a consequence of differences in how blood glucose was measured and in the demographic composition of the study populations.

The HGI could have a practical clinical role in personalizing both hypoglycaemia prevention in diabetes patients and guiding treatment to limit chronic vascular disease in both nondiabetic and diabetic people. Because the HGI measures bias in the quantitative relationship between HbA1c and blood glucose, it could also have a clinical role in diagnosing diabetes when diagnoses were based on HbA1c and blood glucose disagree.^15^ Lack of a standardized way to calculate the HGI in diverse human populations poses a significant barrier to both HGI research and the possible clinical use of the HGI. Widespread standardization would require (1) using assays that give the same HbA1c and glucose results for the same blood sample, and (2) a demographically diverse reference population. We accept that national and international glycated haemoglobin standardization programmes make analytical variation in HbA1c measurement a minor concern. And although there are more comprehensive blood glucose metrics, fasting plasma glucose (FPG) is the simplest, lowest cost and most clinically practical way to assess blood glucose status, especially in economically disadvantaged areas of the world. Furthermore, there is no evidence that the HGI calculated using mean blood glucose, glycated albumin or other measures of blood glucose status provides superior information about risk compared with the HGI calculated using FPG.^1^, ^15^

This report provides rationale for widespread adoption of a simple linear regression equation that can be used to calculate HGI for both research and clinical use. To develop this equation, we first selected a diabetes treatment–naïve reference population from among participants in the 1999–2016 cohorts of the U.S. National Health and Nutrition Examination Survey (NHANES) because NHANES data (1) are carefully collected, comprehensive and publicly available, (2) include FPG and standardized HbA1c measurements and (3) demographically reflect a diverse cross section of the U.S. population. We compared (1) linear regression equations for HbA1c vs. FPG between subgroups of NHANES participants; (2) mean HGI in participants with and without diabetes, iron deficiency and other biochemical, clinical and demographic characteristics; and (3) mean values for selected characteristics in low‐, moderate‐ and high‐HGI participants including those diagnosed as normal, prediabetic or diabetic based on oral glucose tolerance tests (OGTTs). Better understanding of which characteristics are similar between individuals with a low or high HGI and which differ could help identify underlying mechanisms and new ways to prevent hypoglycaemia and chronic vascular disease in people with a high HGI.

## 2 METHODS

### 2.1 Data source

NHANES is an ongoing national survey directed by the Centers for Disease Control that uses a stratified multistage probability sampling design to represent the noninstitutionalized U.S. civilian population.^16^ The National Centers for Health Statistics (NCHS) Ethics Review Board approved the NHANES study protocol, and each participant provided written informed consent.

### 2.2 Study design

Our strategy was to first determine what regression equation to use by assessing the effects of specific inclusion criteria on the slopes, intercepts and coefficients of determination (*r* ^2^) of regression equations describing the linear relationship between FPG and HbA1c. After excluding participants <20 years and those with a self‐reported history of diabetes or taking diabetes medications, the remaining 18,675 diabetes treatment–naïve adult NHANES participants served as a reference population from which to derive a linear regression equation (predicted HbA1c = 0.024 FPG + 3.1) that was used to calculate the HGI in all further analyses. The HGI was calculated by first entering each participant's FPG into the reference population regression equation and then subtracting the resulting predicted HbA1c from the participant's observed HbA1c.

To assess whether other NHANES participants should be excluded from the reference population, we determined the prevalence and mean HGI of subgroups of diabetes treatment–naïve adult participants with selected clinical characteristics that prior research suggests are associated with bias in the quantitative relationship between HbA1c and blood glucose concentration. These include classification based on body mass index (BMI) as normal (<25 kg/m^2^), overweight (25 to <30 kg/m^2^) or obese (≥30 kg/m^2^); insulin resistance based on a HOMA‐IR ≥ 2.5 (HOMA‐IR = Insulin µU/ml × FPG mg/dl)/405^17^; untreated diabetes diagnosed based on American Diabetes Association (ADA)–recommended cut points for FPG (≥126 mg/dl), HbA1c (≥6.5%) or 2 h OGTT (≥200 mg/dl)^18^; anaemia based on World Health Organization (WHO) haemoglobin cut points for men (<13.0 g/dl) and women (<12.0 g/dl)^19^; iron deficiency based on a ferritin <15 µg/l^20^; acute infection based on the white blood cell count (WBC) > 11x10^9^/l^16^; and inflammation based on C‐reactive protein (CRP) classification as low <0.10 mg/dl, average 0.10–0.30 mg/dl or high >0.30 mg/dl.^21^ Statin users were identified as participants taking any of the following cholesterol‐lowering drugs in the past 30 days for which they needed a prescription: simvastatin, lovastatin, atorvastatin, rosuvastatin, pravastatin or pitavastatin. Glucocorticoid users were identified as participants taking any of the following anti‐inflammatory corticosteroids in the past 30 days for which they needed a prescription: prednisone, hydrocortisone, prednisolone, methylprednisolone or dexamethasone.

### 2.3 HGI classification

The diabetes treatment–naïve adult population was divided into tertile (33.3%) subgroups with a low (<−0.150%), moderate (−0.150% to <0.150%) or high (≥0.150%) HGI. We then compared means of selected biochemical, clinical and demographic variables in low‐, moderate‐ and high‐HGI participants in the population as a whole and after further subdivision by diabetes classification based on 2‐h OGTT cut points recommended by the ADA for classification as normal (<140 mg/dl), prediabetic (140 to <200 mg/dl) or diabetic (≥200 mg/dl). The reference population regression equation was also used to calculate HGI for participants who were excluded from the reference population including youth 12–19 years of age and self‐reported diabetic adults.

### 2.4 Statistical analysis

SAS software (Windows version 9.4; SAS Institute) and R (Version 3.3.2; R Core Team) was used for all statistical analyses and data representations. Descriptive statistics were used to characterize the study population. Categorical variables were summarized as frequencies, whereas continuous variables were summarized using means and standard deviations. Log transformations were performed for CRP. Analysis of variance was used to compare means among low‐, moderate‐ and high‐HGI participants. Because statistical significance may not reflect a meaningful biological or clinical significance when applied to very large study populations,^22^ our interpretation of the results also considered whether (1) there was an expected progressive stepwise increase or decrease in the mean of a variable going from the low‐ to the moderate‐ to the high‐HGI subgroup; (2) the magnitude of the difference between low‐ and high‐HGI subgroups was biologically or clinically important based on our best judgement; and (3) an association between a variable and HGI had been previously reported.

## 3 RESULTS

### 3.1 Comparing linear regression equations

Table 1 compares the slopes, intercepts and *r* ^2^ of regression equations describing the linear relationship between HbA1c and FPG in selected subgroups of the combined 1999–2016 NHANES cohorts. HbA1c and FPG data were both available from 28,396 participants, 6790 (23.9%) of whom were 12–19 years of age. Of the 21,606 adult participants, 412 did not answer yes or no when asked if they had a history of diabetes or taking diabetes medications and were excluded from the study. Of the remaining 21,194 adult participants, 2519 (11.9%) were classified as diabetic based on a self‐reported history of diabetes or taking diabetes medications. Of the remaining 18,675 diabetes treatment–naïve adult participants, 10,488 (56.2%) had 2‐h OGTT data collected during morning clinic visits during the years 2005 to 2016.

**TABLE 1:** Comparison of the linear relationship between FPG and HbA1c in subgroups of the combined 1999–2016 NHANES cohorts

Population	*n*	Slope (95% CI)	Intercept (95% CI)	*r* ^2^
HbA1c and FPG results available	28,396	0.025 (0.024, 0.025)	3.02 (3.00, 3.04)	0.680
Youth (age 12–19 years)^a^	6790	0.016 (0.016, 0.017)	3.66 (3.62, 3.71)	0.404
Adult (age ≥ 20 years)	21,606	0.025 (0.025, 0.025)	3.03 (3.01, 3.06)	0.689
Self‐reported diabetic adult^a^, ^b^	2519	0.020 (0.019, 0.021)	4.24 (4.12, 4.36)	0.563
Diabetes treatment–naïve adult	18,675	0.024 (0.023, 0.024)	3.10 (3.07, 3.13)	0.554
Diabetes treatment–naïve adult with OGTT^c^	10,488	0.023 (0.023, 0.024)	3.13 (3.09, 3.18)	0.505

^a^ Excluded from the reference population.

^b^ Self‐reported history of diabetes or taking diabetes medications (412 participants did not answer yes or no and were excluded from the study).

^c^ Study years 2005–2016.

**FIGURE 1:**
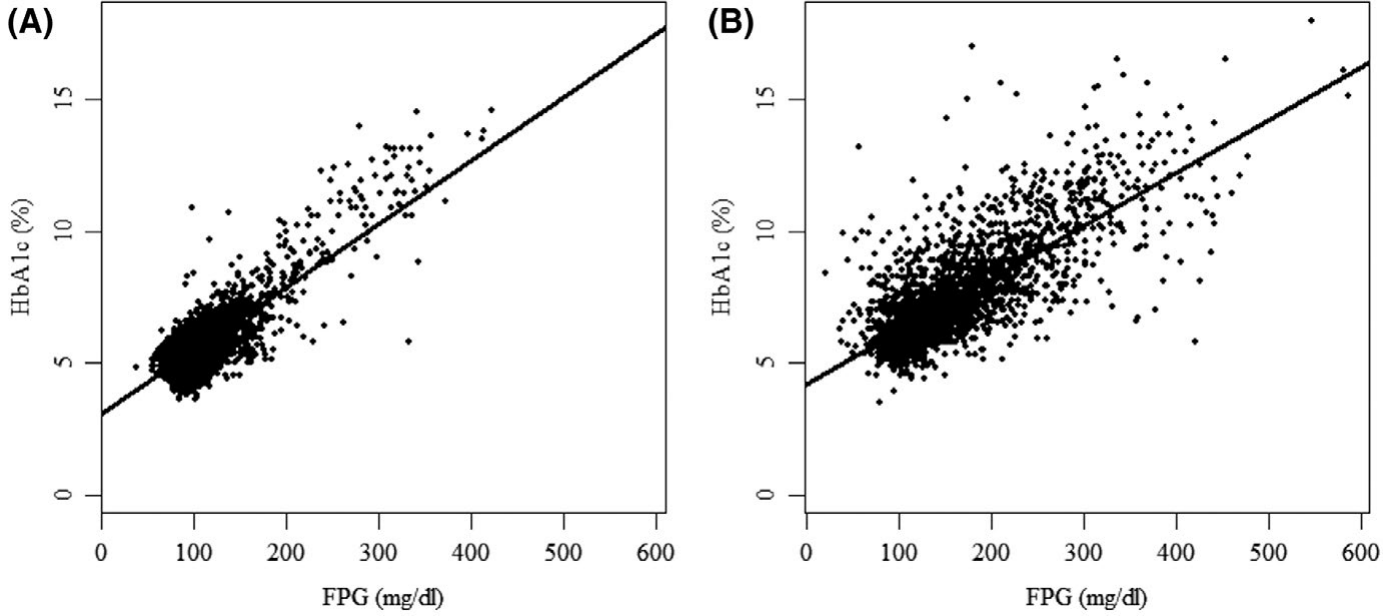
Scatterplots and linear regression parameters for HbA1c vs. FPG in diabetes treatment–naïve adult (Panel A) and self‐reported diabetic adult (Panel B) NHANES participants

**FIGURE 2:**
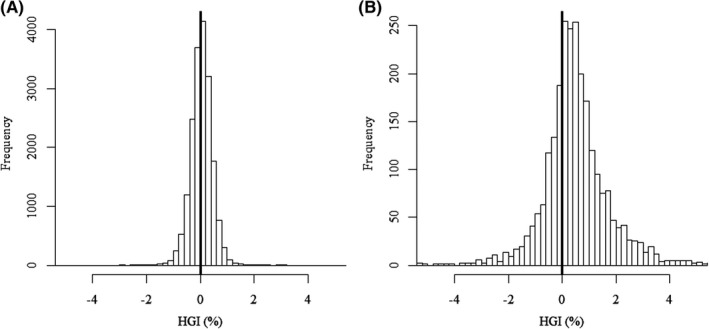
HGI was approximately normally distributed in both diabetes treatment–naïve adult (Panel A) and self‐reported diabetic adult (Panel B) NHANES participants

Table 1 shows that the slopes were lower and intercepts higher for both the 12‐ to 19‐y‐old age‐group (0.016, 3.7) and self‐reported diabetic adults (0.020, 4.2) than for diabetes treatment–naïve adult participants (0.024, 3.1). Figure 1 graphically compares the distribution of paired HbA1c and FPG observations from diabetes treatment–naïve adult participants (Panel A) with that from self‐reported diabetic adults (Panel B). Figure 2 shows that HGI calculated using the reference population regression equation was approximately normally distributed in both the diabetes treatment–naïve adult (Panel A) and self‐reported diabetic adult (Panel B) populations. However, diabetic adults had a markedly higher mean HGI (+0.577%) and an HGI range that was three times greater than that of diabetes treatment–naïve adults. The mean HGI for 12‐ to 19‐y‐old NHANES participants was −0.092% ± 0.370.

### 3.2 Characteristics associated with HGI

Table 2 catalogues the mean HGI and prevalence of diabetes treatment–naïve adult NHANES participants after subdivision into groups with selected clinical characteristics. Table 3 compares means for selected biochemical, clinical and demographic variables in the diabetes treatment–naïve adult population as a whole, and when divided into low (<−0.150)‐, moderate (−0.150 to <0.150)‐ and high (≥0.150)‐HGI subgroups. Because of how HGI is calculated, dividing the reference population into HGI tertiles naturally produces roughly equal‐sized subgroups with similar mean FPG but progressively higher mean HbA1c and HGI going from the low‐ to moderate‐ to high‐HGI subgroup. A higher mean HGI was associated with trends for older age, higher BMI and higher CRP, plus disproportionately more female individuals and blacks. There was a slight inverse trend between the mean HGI and haemoglobin concentration but no biologically relevant positive or negative trends in plasma insulin, HOMA‐IR or 2‐h OGTT.

**TABLE 2:** Mean HGI and prevalence of NHANES reference population participants with selected clinical characteristics

Population	Characteristic	*n*	Prevalence %^a^	HGI Mean ± SD
Diabetes treatment–naïve adults	≥20 years, No self‐reported history of diabetes	18,675	‐	0.000 ± 0.41
Normal weight	BMI < 25	6051	32.9	−0.028 ± 0.38
Overweight	BMI 25 to <30 kg/m^2^	6395	34.7	−0.011 ± 0.42
Obese	BMI ≥ 30	5959	32.4	0.049 ± 0.43
Insulin‐resistant	HOMA‐IR ≥ 2.5	8196	44.7	0.002 ± 0.47
Diabetes treatment–naïve with diabetes	FPG ≥ 126 mg/dl	841	4.5	−0.010 ± 0.92
	HbA1c ≥ 6.5%	514	2.8	0.760 ± 0.81
	2 h OGTT ≥200 mg/dl	684	6.5^b^	0.170 ± 0.67
Anaemia	Hb < 13.0 g/dl – men	1489	8.0	−0.082 ± 0.47
	Hb < 12.0 g/dl – women			
Iron‐deficient	Ferritin < 15 μg/l	729	12.4^c^	−0.030 ± 0.35
Asthma	Self‐reported questionnaire	2391	12.8	0.004 ± 0.42
Acute infection	WBC > 11 x 10^9^/l	676	3.6	0.020 ± 0.42
Statin use^d^	Self‐reported questionnaire	2177	11.7	0.110 ± 0.42
Glucocorticoid use^e^	Self‐reported questionnaire	218	1.2	0.140 ± 0.49

^a^ Among the 18,675 diabetes treatment–naïve adult participants in study years 1999–2016 unless otherwise noted.

^b^ Among the subgroup of 10,488 participants with OGTT results (years 2005–2016).

^c^ Study years 1999–2002, 2005–2008 and 2015–2016.

^d^ Participants using any of the cholesterol lowering drugs listed in methods.

^e^ Participants using any of the anti‐inflammatory corticosteroids listed in methods.

**TABLE 3:** Mean values for selected characteristics in the diabetes treatment–naïve adult NHANES reference population as a whole and when divided into HGI tertile subgroups

Characteristic	All	Low <−0.150	Moderate −0.150 to <0.150	High ≥0.150	*p*
*N*	18,675	6210	6227	6238	‐
Reference population (%)	‐	33.3	33.3	33.4	‐
HGI (%)	0.000	−0.420	0.003	0.420	<.001
HbA1c (%)	5.5	5.1	5.4	5.9	<.001
FPG (mg/dl)	100	103	98	99	<.001
Age (years)	48	44	47	52	<.001
BMI (kg/m^2^)	28.3	27.8	28.1	29.1	<.001
CRP (mg/dl)^a^, ^b^	0.46	0.43	0.43	0.54	<.001
Female, *n* (%)	9766 (52)	2863 (46)	3357 (54)	3546 (57)	<.001
Black, *n* (%)	3510 (19)	778 (13)	950 (15)	1782 (29)	<.001
Haemoglobin (g/dl)	14.1	14.2	14.1	13.9	<.001
Insulin (µU/ml)	12.5	13.3	11.5	12.7	<.001
HOMA‐IR	3.2	3.6	2.9	3.3	<.001
2‐h OGTT (mg/dl)^c^	120	116	115	128	<.001

^a^ Study years 1999–2010.

^b^ Log‐transformed.

^c^ Study years 2005–2016.

Table 4 compares the same characteristics in the subgroup of diabetes treatment–naïve adult participants that have OGTT data after further subdivision into normal, prediabetic and diabetic subgroups based on the OGTT. The proportion of participants classified as low (33.5%), moderate (34.0%) or high (32.5%) HGI was similar in the 76.3% of the population diagnosed as normal by OGTT. In contrast, there were 10% more high HGI participants in the 17.2% of the population diagnosed as prediabetic, and over 20% more high‐HGI participants in the 6.5% of the population diagnosed as diabetic.

**TABLE 4:** Mean values for selected characteristics in diabetes treatment–naïve adult NHANES participants with OGTT data (study years 2005–2016) divided by both HGI subgroup and diabetes classification based on ADA OGTT cut points

Normal (76.3%) 2‐h OGTT < 140 mg/dl	All	Low <−0.150	Moderate −0.150 to <0.150	High ≥0.150	*p*
*n*	8000	2679	2722	2599	‐
Normal population (%)	‐	33.5	34.0	32.5	‐
HGI (%)	−0.010	−0.410	0.000	0.380	<.001
HbA1c (%)	5.4	5.0	5.4	5.7	<.001
FPG (mg/dl)	97	100	97	95	<.001
Age (years)	45	40	44	50	<.001
BMI (kg/m^2^)	28.0	27.8	27.8	28.4	<.001
CRP (mg/dl)^a^	0.36	0.33	0.35	0.42	<.001
Female, *n* (%)	4034 (50)	1164 (43)	1411 (52)	1459 (56)	<.001
Black, *n* (%)	1549 (19)	340 (13)	424 (16)	785 (30)	<.001
Haemoglobin (g/dl)	14.2	14.6	14.3	13.9	<.001
Insulin (µU/ml)	11.1	11.5	10.8	11.1	.008
HOMA‐IR	2.7	2.9	2.6	2.6	<.001
2‐h OGTT (mg/dl)	99	98	98	100	.005
Prediabetic (17.2%) 2‐h OGTT 140 to <200 mg/dl					
*n*	1804	536	552	716	‐
Prediabetic population (%)	‐	29.7	30.6	39.7	‐
HGI (%)	0.040	−0.440	0.000	0.430	<.001
HbA1c (%)	5.6	5.2	5.6	5.9	<.001
FPG (mg/dl)	106	110	105	103	<.001
Age (years)	57	54	56	59	<.001
BMI (kg/m^2^)	30.2	29.4	29.9	31.0	.002
CRP (mg/dl)^a^, ^b^	0.58	0.49	0.58	0.66	.035
Female, *n* (%)	916 (51)	240 (45)	269 (49)	407 (57)	<.001
Black, *n* (%)	264 (15)	50 (9)	55 (10)	159 (22)	<.001
Haemoglobin (g/dl)	13.7	13.8	13.9	13.6	.450
Insulin (µU/ml)	15.3	16.0	14.7	15.1	.314
HOMA‐IR	4.0	4.4	3.9	3.9	.167
2‐h OGTT (mg/dl)	162	162	161	164	.048
Diabetic (6.5%) 2‐h OGTT ≥ 200 mg/dl					
*n*	684	184	168	332	‐
Diabetic population (%)	‐	26.9	24.6	48.5	‐
HGI (%)	0.17	−0.53	0.01	0.64	<.001
HbA1c (%)	6.4	5.7	6.0	7.0	<.001
FPG (mg/dl)	134	134	123	139	.013
Age (years)	62	63	61	61	.425
BMI (kg/m^2^)	30.8	30.1	31.1	31.0	.334
CRP (mg/dl)^a^, ^b^	0.74	0.74	0.67	0.77	.057
Female, *n* (%)	339 (50)	71 (39)	91 (54)	177 (53)	.001
Black, *n* (%)	90 (13)	17 (9)	15 (9)	58 (17)	.009
Haemoglobin (g/dl)	14.4	14.7	14.4	14.3	.022
Insulin (µU/ml)	17.8	17.6	17.8	17.9	.415
HOMA‐IR	6.0	6.1	5.8	6.1	.411
2‐h OGTT (mg/dl)	256	249	237	269	<.001

^a^ Study years 2005–2010.

^b^ Log‐transformed.

Mean age progressively increased going from low to moderate to high HGI in diabetes treatment–naïve adult participants diagnosed as normal or prediabetic but not in those diagnosed as diabetic. The difference in mean age between low‐ and high‐HGI participants was markedly greater in those diagnosed as normal (+10 years) than in those diagnosed as prediabetic (+5 years) or diabetic (−2 years). Mean age progressively increased within each HGI subgroup going from normal to prediabetic to diabetic. The range in mean age between normal and diabetic subgroups was markedly greater in low‐HGI participants (23 years) than that observed in moderate (17 years)‐ or high (11 years)‐HGI participants.

Most biochemical, clinical and demographic observations in the normal and prediabetic subgroups (Table 4) were similar to those observed in the diabetes treatment–naïve adult NHANES population as a whole (Table 3). This includes (1) similar mean FPG between HGI subgroups; (2) progressively higher HbA1c, older age, higher BMI and higher CRP, as well as disproportionately more female individuals and blacks going from the low‐ to moderate‐ to high‐HGI subgroup; and (3) no consistent positive or negative trends in plasma insulin, HOMA‐IR or 2 h OGTT across HGI subgroups. Trends for more female individuals and blacks with increasing mean HGI were also observed in diabetic participants, but trends for older age and higher BMI were not.

Mean CRP increased and approximately doubled within all three HGI subgroups going from normal to prediabetic to diabetic classification. Plasma insulin, HOMA‐IR and 2 h OGTT also progressively increased within all three HGI subgroups with worsening diabetes status. The inverse trend observed between haemoglobin concentration and mean HGI in the diabetes treatment–naïve adult population as a whole (Table 3) was more pronounced in participants classified as normal by OGTT but not in participants with prediabetes or diabetes (Table 4).

## 4 DISCUSSION

### 4.1 Quantifying bias in the quantitative relationship between HbA1c and blood glucose

The HGI was first proposed in 2002 as a way to quantify how far an individual's observed HbA1c lies above or below average compared with other people with similar blood glucose concentrations.^1^ Since then, it has become increasingly clear that bias in the quantitative relationship between HbA1c and blood glucose has important clinical implications for the diagnosis and management of diabetes and chronic vascular disease. The FPG‐based HGI and two related metrics called the glycation gap (which is based on glycated albumin^23^, ^24^) and the glucose management indicator (which is based on mean blood glucose [MBG]^25^) were recently reviewed by Nayak et al.^26^ We propose that FPG should be the metric of choice for standardizing HGI because unlike MBG or glycated albumin, FPG is a simple, reliable, low‐cost clinical test that is readily available worldwide. One drawback to the use of FPG is that patient failure to comply with the fasting directive could produce a falsely low HGI. We used HbA1c results expressed as a percentage of total haemoglobin and FPG in mg/dl to be consistent with most prior HGI research. The demographically diverse NHANES study population was used to derive the reference population because of the remarkable quantity, quality and accessibility of NHANES data. Analyses were restricted to the 1999 or later NHANES cohorts because HbA1c assays were not all standardized in earlier cohorts.

### 4.2 Selection of the diabetes treatment–naïve adult reference population

The first issue we addressed was whether any NHANES participants should be excluded from the reference population used to derive the standard HGI regression equation, and if so, what criteria should be used and why? We chose to exclude participants <20 years mainly to be consistent with prior studies. When calculated using the proposed standard HGI regression equation, the mean HGI for the 12‐ to 19‐y‐old subgroup of NHANES participants was −0.092% lower than that of the diabetes treatment–naïve adult population. A relationship between older age and higher HGI is apparent in Tables 3 and 4 which show clear trends towards older age with a higher mean HGI in the diabetes treatment–naïve adult population as a whole and in normal and prediabetic participants, but not in diabetic participants. These observations are consistent with multiple reports of an association between higher HGI and older age in nondiabetic study populations^2^, ^3^, ^16^ but not diabetic study populations where age either did not differ^10^, ^11^, ^14^, ^27^, ^28^, ^29^, ^30^ or was higher in the low‐HGI subgroup.^8^, ^12^, ^13^

We also excluded participants with a self‐reported history of diabetes or taking diabetes medications because the present study and published research conclusively show that the quantitative relationship between HbA1c and FPG in people treated for diabetes differs from that typically observed in people without diagnosed diabetes. Table 1 and Figure 1 show that the regression equation for NHANES participants with a self‐reported history of diabetes had a lower slope and a higher intercept (0.020, 4.2) than the diabetes treatment–naïve adult population (0.024, 3.1). It is important to note that among twelve studies of HGI in type 2 diabetes populations,^8^, ^9^, ^10^, ^11^, ^12^, ^13^, ^14^, ^27^, ^28^, ^29^, ^30^, ^31^ all had lower slopes (mean 0.017, range 0.008 to 0.022) and higher intercepts (mean 5.2, range 4.4–6.8) than the diabetes treatment–naïve adult NHANES population. Collectively, these results show that diabetes is associated with an abnormal quantitative relationship between HbA1c and blood glucose compared with what was observed in diabetes treatment–naïve adult NHANES participants.

### 4.3 Characterization of the diabetes treatment–naïve adult reference population

Table 2 shows that obesity was associated with a small but progressive increase in the mean HGI going from normal (−0.028) to overweight (−0.011) to obese (0.049) subgroups (range 0.077%). Although 44.5% of diabetes treatment–naïve adult participants were classified as insulin‐resistant based on HOMA‐IR, the mean HGI was not different from the population as a whole. Diabetes treatment–naïve adult participants diagnosed as diabetic based on an FPG ≥ 126 mg/dl had a mean HGI that was not different from that observed in the population as a whole. In contrast, participants diagnosed as diabetic based on HbA1c ≥ 6.5% had a + 0.760% higher mean HGI. That HGI is higher in untreated adults with diabetes is graphically supported by Figure 1A which shows that nearly all diabetes treatment–naïve adults with FPG over 175 mg/dl had HbA1c above the population regression line: the hallmark of a high HGI phenotype.

Insulin resistance and impaired glucose tolerance (IGT) are among the first traits to emerge as people transition from a normal to a diabetic metabolic state. It is a biochemical fact of nonenzymatic haemoglobin glycation that with all other conditions equal, chronically higher FPG or postprandial glucose will increase the rate of HbA1c accumulation in red blood cells (RBCs). Because the HGI is calculated using FPG and because FPG is not elevated in isolated IGT, the onset of persistently higher postprandial glucose levels should naturally result in higher than usual levels of both HbA1c and HGI when calculated using FPG. The mean HGI of 6.5% of diabetes treatment–naïve adults diagnosed as diabetic based on 2‐h OGTT ≥ 200 mg/dl was 0.170% (Table 2), which is over half the 0.300% HGI range observed in moderate‐HGI participants (−0.150 to +0.150) and thus enough to shift someone who usually has an HGI of 0.000% from a moderate‐ to a high‐HGI classification. A longitudinal study is needed to test the hypothesis that an increase in a previously undiagnosed person's usual HGI is diagnostic of new‐onset diabetes and thus clinical rationale for a confirmatory OGTT.

Although population variation in postprandial glucose undoubtedly contributes to population variation in HbA1c and HGI, there were no biologically significant positive or negative trends in 2‐h OGTT glucose levels in low‐, moderate‐ or high‐HGI diabetes treatment–naïve adult participants as a whole (Table 3) or when subdivided into normal, prediabetic and diabetic subgroups by OGTTs (Table 4). Lack of association between the HGI and 2‐h OGTT in the present study and in three previous studies^2^, ^15^, ^32^ strongly suggests that population variation in the HGI based on FPG is not an artefact of person‐to‐person variation in postprandial blood glucose concentration.

Haemolytic anaemia shortens the RBC lifespan, and the amount of time HbA1c has to accumulate inside RBCs.^33^ Consequently, with all other conditions equal, a shorter RBC lifespan will naturally lower both HbA1c and HGI. In the present study, NHANES participants with low haemoglobin levels diagnostic of anaemia represented 8.0% of the diabetes treatment–naïve adult population (Table 2) and had a mean HGI that was −0.082% lower than that observed in the overall population. In contrast however, mean haemoglobin concentration was higher in low‐HGI participants than high‐HGI participants (Tables 3 and 4), which is opposite of what one would expect if anaemia was responsible for population variation in the HGI. Thus, although a shortened RBC lifespan can lower an individual's HGI, anaemia alone does not explain the range of the HGI observed in the diabetes treatment–naïve adult NHANES population.

HbA1c is reportedly higher than normal in people with iron deficiency^34^ or asthma^35^ independent of blood glucose concentration, which should produce a higher than normal HGI. Table 2 shows, however, that although participants with iron deficiency or asthma were relatively prevalent in the population (12.4 and 12.8%, respectively), their mean HGI was similar to that observed in the diabetes treatment–naïve adult population as a whole. The mean HGI was also not different in participants with acute infection based on the WBC count. Higher than normal HbA1c levels have been reported in statin users^36^ and glucocorticoid users^37^ independent of blood glucose concentration. The observation that the mean HGI was 0.11% higher in statin users and 0.14% higher in glucocorticoid users supports the conclusion that these drugs alter the normal quantitative relationship between HbA1c and blood glucose.

### 4.4 Clinical implications

Longitudinal studies have shown that people with diabetes tend to have an HGI that is significantly different between individuals but relatively consistent within individuals over time and over the physiological range of blood glucose concentrations.^1^, ^8^, ^38^ And there is little doubt that genetic variation is a major source of person‐to‐person variation in HbA1c.^39^, ^40^ The present study adds to a growing list of biological, clinical and demographic factors associated with bias in the quantitative relationship between HbA1c and blood glucose concentration. It is important to note that although the contribution of any one factor to an individual's HGI may be relatively small, combinations of factors that promote low or high HGI could collectively produce individuals with the range of HGIs observed in human populations.

The suitability of the diabetes treatment–naïve adult NHANES participants as the reference population and model for HGI in demographically diverse human populations is supported by the fact that our results confirm what has been consistently reported in the literature, including observations of (1) disproportionately more black participants having a higher HGI independent of diabetes classification; (2) biologically relevant trends between a higher HGI and older age, higher BMI and higher CRP in normal and prediabetic participants, but not in diabetic participants; and (3) lack of association between HGI and 2‐h OGTT results, except possibly in individuals with new‐onset diabetes. The association between a higher HGI and female sex observed in the present study has been reported in some studies^3^, ^6^, ^8^, ^11^, ^32^ but not consistently across studies.

We propose that the simple linear regression equation derived from the carefully curated and demographically diverse diabetes treatment–naïve adult NHANES reference population can be used to standardize the HGI for research and clinical use. The use of FPG makes calculating the HGI an easily automatable part of a hospital's electronic health record system. It also makes it easy for health‐care providers in remote locations to calculate HGI manually. The use of FPG also makes it possible to retrospectively study the HGI phenomenon in ongoing or completed clinical trials that collected FPG and standardized HbA1c data. Standardizing how HGI is calculated will facilitate both clinical implementation of HGI and basic research to explain (1) why some people have consistently lower or higher than average HbA1c levels than other people with similar blood glucose concentrations, (2) why a high HGI phenotype is associated with greater risk for chronic vascular disease, and (3) what can be done to reduce risk in high‐HGI people.

## CONFLICT OF INTEREST

The authors have no conflicts to disclose.

## AUTHOR CONTRIBUTION

**James M. Hempe:** Conceptualization (lead); Investigation (equal); Methodology (equal); Project administration (equal); Supervision (equal); Writing–original draft (lead); Writing–review and editing (equal). **Shengping Yang:** Conceptualization (equal); Data curation (lead); Formal analysis (lead); Investigation (equal); Methodology (equal); Software (equal); Validation (equal); Writing–review and editing (equal). **Shuqian Liu:** Formal analysis (equal); Investigation (equal); Methodology (equal); Validation (equal); Writing–review and editing (equal). **Daniel S. Hsia:** Investigation (equal); Methodology (equal); Project administration (equal); Supervision (equal); Validation (equal); Writing–review and editing (equal).

## Data Availability

The data that support the findings of this study are openly available through the National Health and Nutrition Examination Survey at https://wwwn.cdc.gov/nchs/nhanes/.
