# Corrigendum

**DOI:** 10.1002/edm2.322

**Published:** 2022-03-12

In the article by Hempe et al.,^1^ Figures 1 and Figure 2 contain errors.

1. In Figure 1, regression equations are missing in the published version. The correct figure as follows:

**FIGURE 1:**
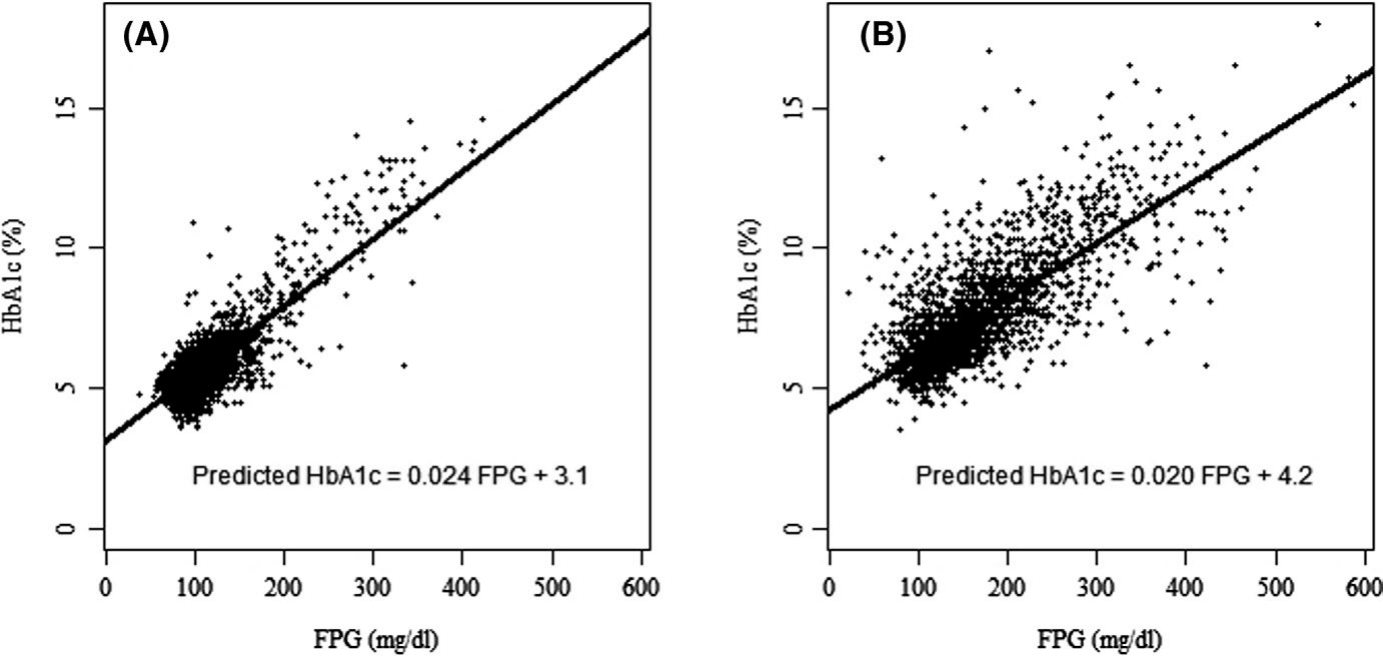
Scatterplots and linear regression parameters for HbA1c vs. FPG in diabetes treatment‐naive adult (Panel A) and self‐reported diabetic adult (Panel B) NHANES participants

2. In Figure 2, Mean and sd values are missing in the published version. The correct figure is as follows:

**FIGURE 2:**
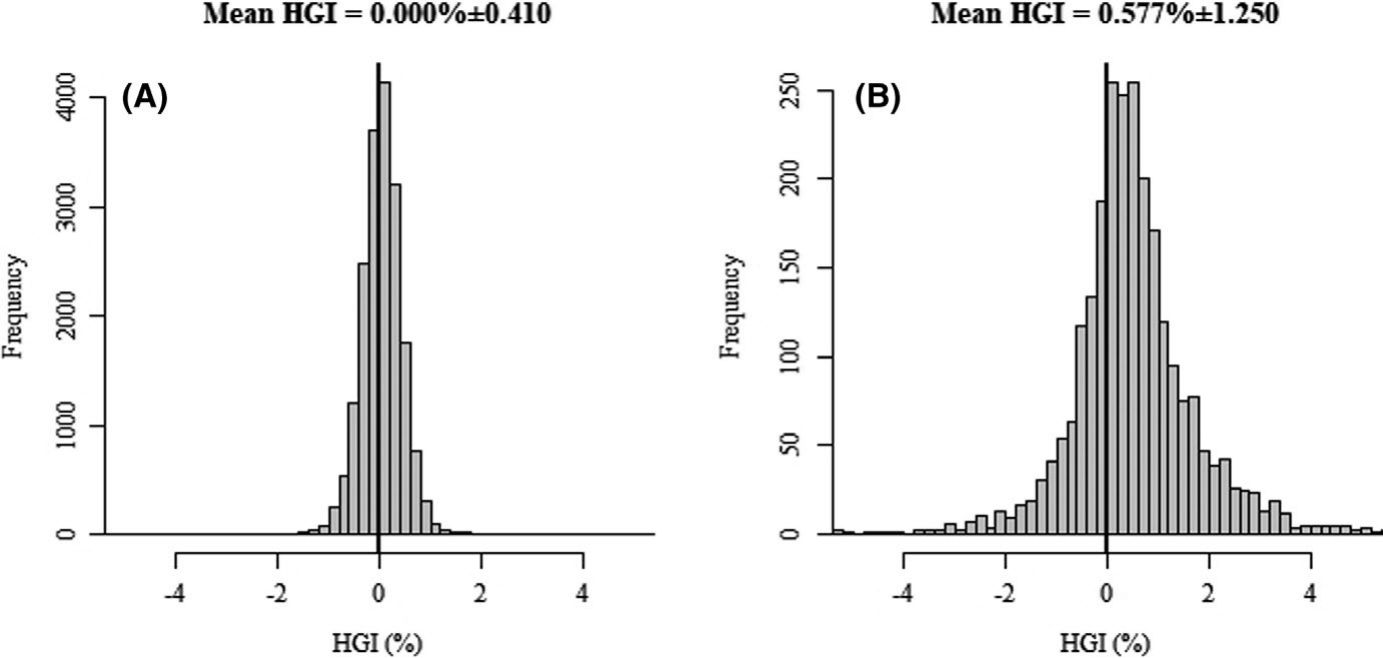
HGI was approximately normally distributed in both diabetes treatment–naive adult (Panel A) and self‐reported diabetic adult (Panel B) NHANES participants

The authors apologize for these errors.
